# Supplementary material for: Insights into Collagen Uptake by C-type Mannose Receptors from the Crystal Structure of Endo180 Domains 1–4
Source: Structure. 2015 Nov 3;23(11):2133–42. doi: 10.1016/j.str.2015.09.004 (PMC4635314; doi:10.1016/j.str.2015.09.004)
Supplement: Document S1. Figures S1–S4 [file mmc1.pdf]

Structure, Volume *23*

Supplemental Information

Insights into Collagen Uptake by C-type Mannose Receptors from the Crystal Structure of Endo180 Domains 1–4

Patricia Paracuellos, David C. Briggs, Federico Carafoli, Tan Lončar, and Erhard Hohenester

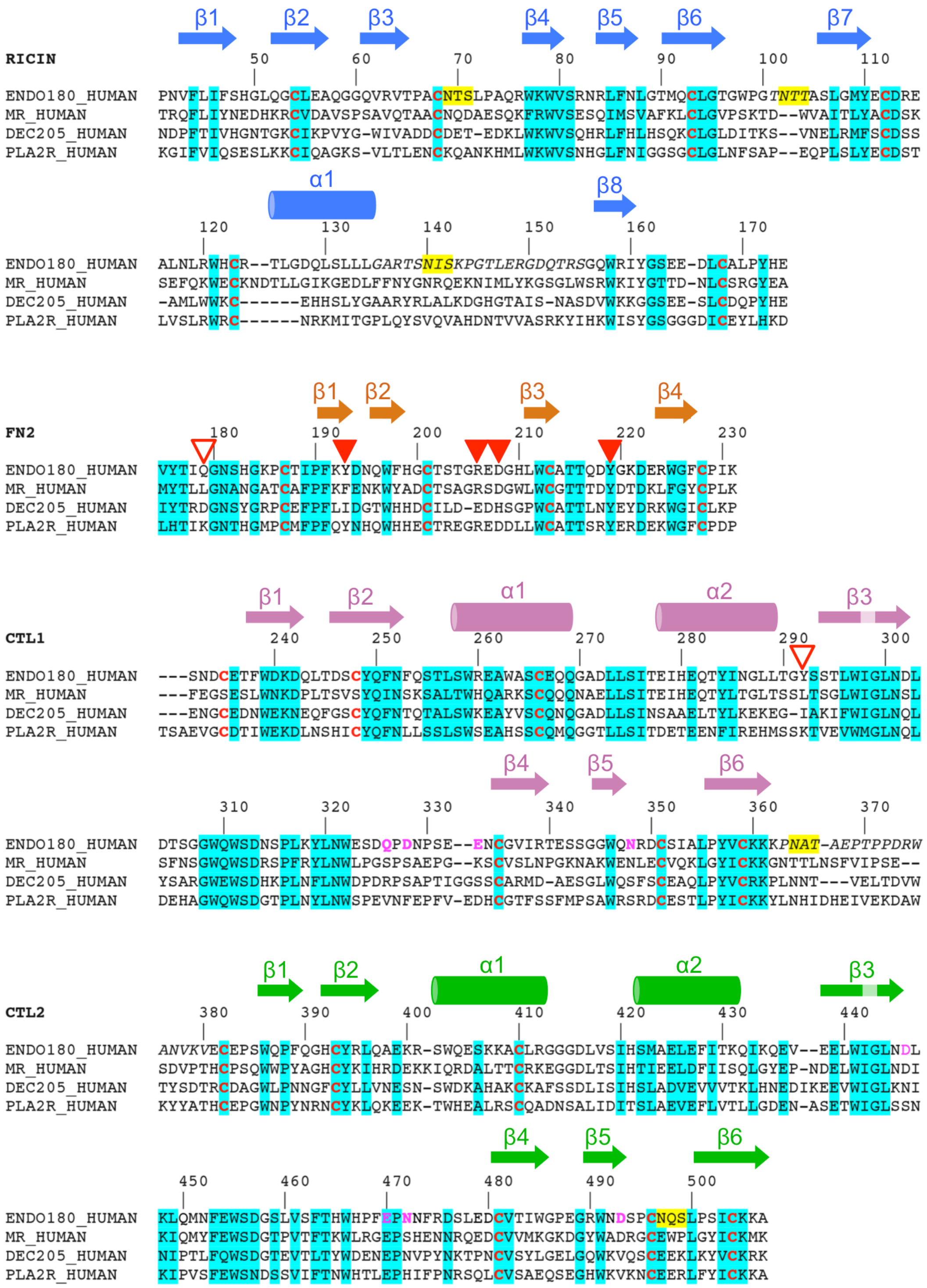

Figure S1, related to Figure 1. Sequence alignment of the D1-4 regions of human MR, Endo180, PLA_2R and DEC-205. The Endo180 sequence numbering and secondary structure elements are indicated above the alignment. The sequences of disordered regions in the Endo180 crystal structure are in italics. Consensus sites for *N*-linked glycosylation are highlighted in yellow. Residues involved in metal ion binding are in magenta. Residues whose mutation to alanine abolishes collagen binding are marked by filled red triangles. Positions at which an engineered glycan reduces collagen binding are marked by open red triangles.

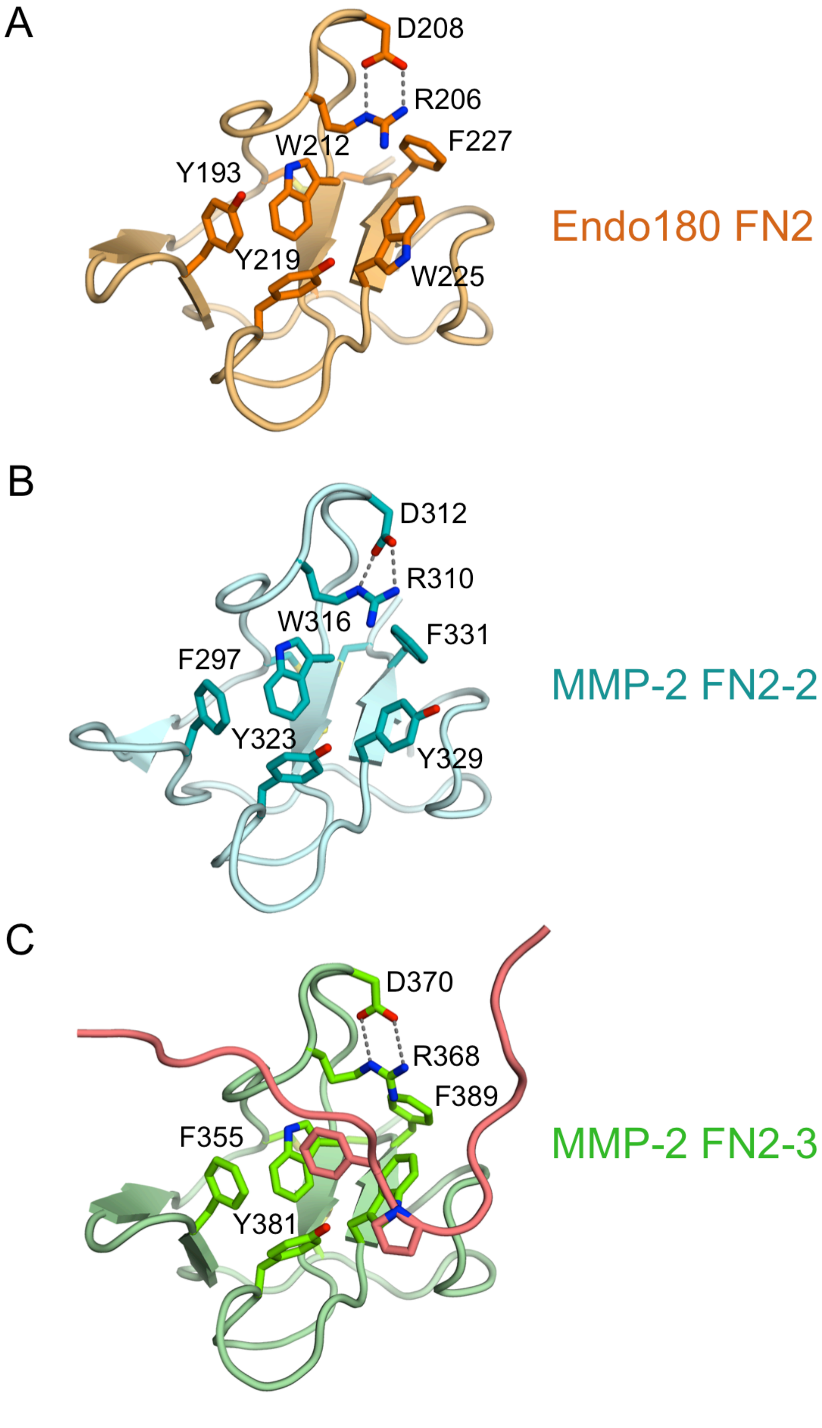

Figure S2, related to Figure 1. Comparison of the FN2 domain of Endo180 and the second and third FN2 domains of MMP-2 (Morgunova et al., 1999). Selected conserved residues are shown as sticks and labelled. In the pro-MMP-2 crystal structure, the propeptide (shown in salmon) interacts with the third FN2 domain in a manner believed to mimic the binding of gelatin.

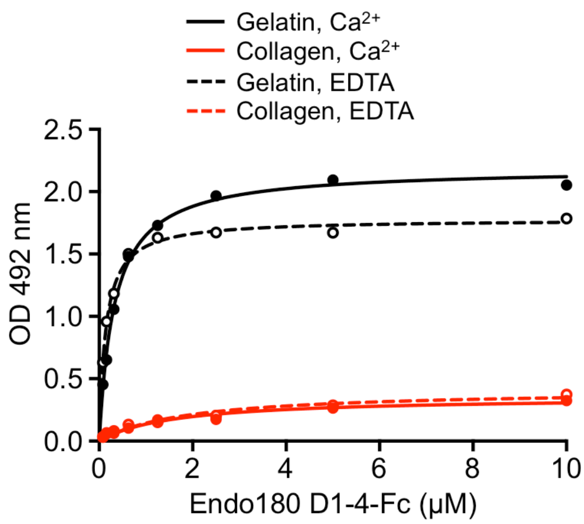

Figure S3, related to Figure 5. Solid-phase assay of Endo180 D1-4-Fc binding to immobilised type I collagen and gelatin (heat-denatured type I collagen) in the presence of 10 mM Ca^{2+} or 10 mM EDTA. The data shown are representative of two independent experiments carried out in duplicate.

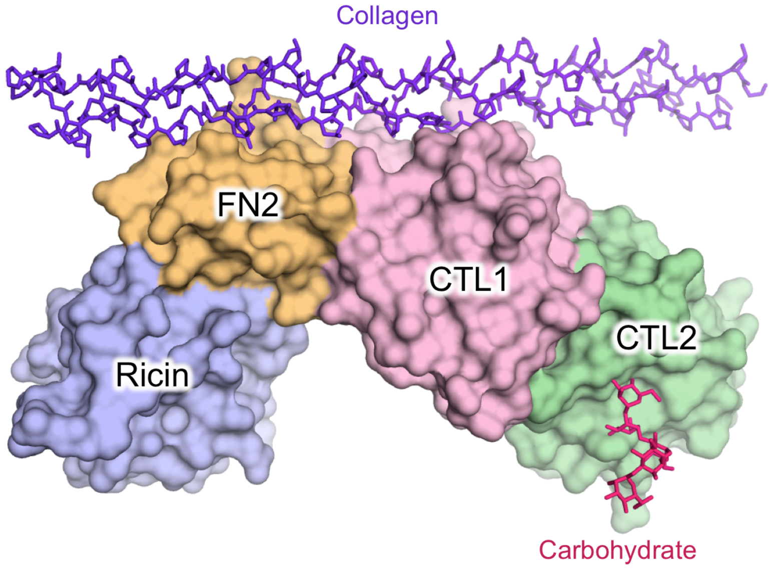

Figure S4, related to Figure 6. Model of a collagen triple helix bound to Endo180 D1-4. An idealised poly-(proline-hydroxyproline-glycine) triple helix was docked using ZDOCK (Pierce et al., 2014). The potential position of carbohydrate bound to CTL2 was obtained from the comparison with mannose-binding protein (Figure 3A).

Reference

Pierce, B.G., Wiehe, K., Hwang, H., Kim, B.H., Vreven, T., and Weng, Z. (2014). ZDOCK server: interactive docking prediction of protein-protein complexes and symmetric multimers. Bioinformatics *30*, 1771-1773.
