# Supplementary material for: Insights into Collagen Uptake by C-type Mannose Receptors from the Crystal Structure of Endo180 Domains 1–4
Source: Structure. 2015 Nov 3;23(11):2133–42. doi: 10.1016/j.str.2015.09.004 (PMC4635314; doi:10.1016/j.str.2015.09.004)
Supplement: Document S2. Article plus Supplemental Information [file mmc2.pdf]

Structure

Article

Insights into Collagen Uptake by C-type Mannose Receptors from the Crystal Structure of Endo180 Domains 1–4

Graphical Abstract

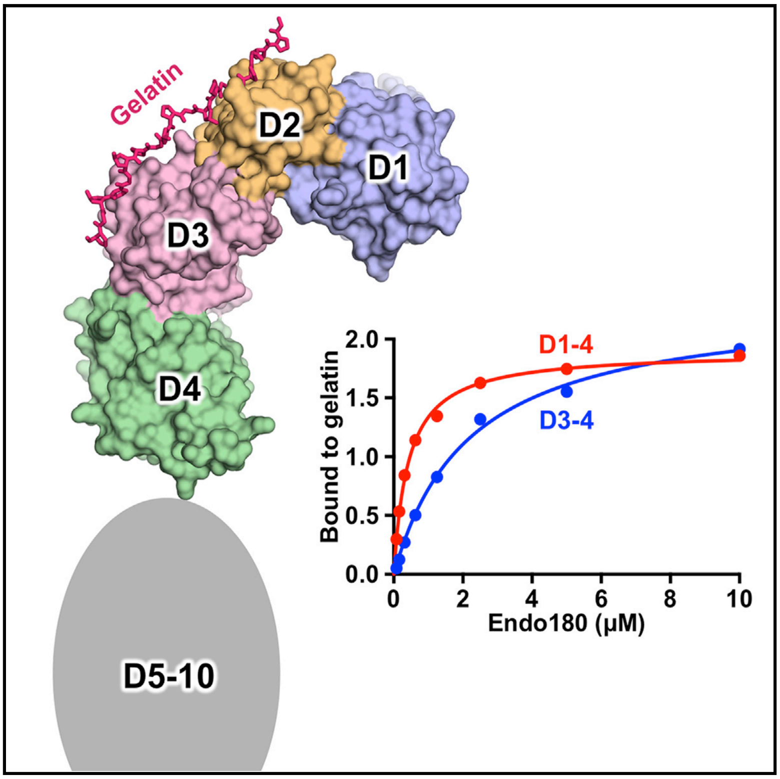

Authors

Patricia Paracuellos, David C. Briggs, Federico Carafoli, Tan Lončar, Erhard Hohenester

Correspondence

e.hohenester@imperial.ac.uk

In Brief

Endo180 is an endocytic receptor that clears collagen from the extracellular space. Paracuellos et al. have determined crystal and solution structures of the collagen-binding head of Endo180 and show that the presumed collagen release in endosomes is not regulated by changes within the head.

Highlights

- Domains 1–4 of the endocytic receptor Endo180 form an L-shaped structure
- The fibronectin type II domain (domain 2) is crucial for collagen/gelatin binding
- The first C-type lectin domain (domain 3) also contributes to collagen binding
- Collagen binding to Endo180 domains 1–4 is not reduced by low pH or Ca^{2+} depletion

Accession Numbers

5AO5
5AO6

Paracuellos et al., 2015, Structure *23*, 2133–2142
November 3, 2015 ©2015 The Authors
http://dx.doi.org/10.1016/j.str.2015.09.004

CellPress

Insights into Collagen Uptake by C-type Mannose Receptors from the Crystal Structure of Endo180 Domains 1–4

Patricia Paracuellos,[1,2] David C. Briggs,[1,2] Federico Carafoli,[1] Tan Lončar,[1] and Erhard Hohenester[1,*]
[1]Department of Life Sciences, Imperial College London, London SW7 2AZ, UK
[2]Co-first author
*Correspondence: e.hohenester@imperial.ac.uk
http://dx.doi.org/10.1016/j.str.2015.09.004
This is an open access article under the CC BY license (http://creativecommons.org/licenses/by/4.0/).

SUMMARY

The C-type mannose receptor and its homolog Endo180 (or uPARAP, for urokinase plasminogen activator receptor-associated protein) mediate the endocytic uptake of collagen by macrophages and fibroblasts. This process is required for normal tissue remodeling, but also facilitates the growth and dissemination of tumors. We have determined the crystal structure at 2.5 Å resolution of the N-terminal region of Endo180, consisting of a ricin-like domain, a fibronectin type II (FN2) domain, and two C-type lectin (CTL) domains. The L-shaped arrangement of these domains creates a shallow trench spanning the FN2 and CTL1 domains, which was shown by mutagenesis to bind triple-helical and denatured collagen. Small-angle X-ray scattering showed that the L-shaped structure is maintained in solution at neutral and acidic pH, irrespective of calcium ion loading. Collagen binding was equally unaffected by acidic pH, suggesting that collagen release in endosomes is not regulated by changes within the Endo180 N-terminal region.

INTRODUCTION

Collagens are a major component of extracellular matrix and are essential for tissue stability. Fibrillar collagens, such as type I collagen, are found in interstitial matrices and form highly ordered suprastructures in the cornea, skin, tendon, and bone. The non-fibrillar type IV collagen is an invariant and abundant component of basement membranes (Kadler et al., 2007). Regulated breakdown of collagen is required for morphogenesis, tissue remodeling, and repair. Perturbed collagen homeostasis is associated with a range of diseases, including organ fibrosis, scarring, and arthritis. Furthermore, malignant tumor cells need to breach collagen-rich matrices to disseminate and form metastases (Bonnans et al., 2014). The triple-helical structure and large size of collagens necessitate unique degradation mechanisms. Collagenolytic matrix metalloproteinases (MMPs) initially cleave fibrillar collagens at a single site three-quarters from the N terminus of the triple helix (Fields, 2013). The triple helices of the resulting fragments unfold at body temperature (Danielsen, 1987; Gross and Nagai, 1965), and the denatured collagen fragments are further degraded by a variety of extracellular proteinases or internalized by macrophages and fibroblasts for lysosomal degradation (Everts et al., 1996; Madsen et al., 2011).

The major route of non-phagocytic collagen clearance is through endocytosis by the C-type mannose receptor (MR) and its homolog Endo180 (also called urokinase plasminogen activator receptor-associated protein or uPARAP) (East et al., 2003; Engelholm et al., 2003; Martinez-Pomares et al., 2006; Wienke et al., 2003). Cultured cells expressing MR or Endo180 take up labeled collagen and route it to lysosomal compartments for degradation. Cleaved or heat-denatured collagen (gelatin) is taken up more avidly than intact collagen (Madsen et al., 2007), and this preference is also observed in binding studies with purified MR and Endo180 proteins (Martinez-Pomares et al., 2006; Wienke et al., 2003). The size of fibrillar collagens far exceeds that of typical endocytic vesicles, and it is unclear whether intact collagen actually can be endocytosed. Phagocytosis of collagen fibrils is an established phenomenon, however (Everts et al., 1996).

Mice lacking a functional Endo180 are phenotypically normal, but fibroblasts derived from these animals are incapable of collagen uptake (East et al., 2003; Engelholm et al., 2003). The impaired collagen turnover in Endo180-deficient mice was shown to reduce tumor growth in a mammary adenocarcinoma model (Curino et al., 2005). Analysis of MR-deficient mice is complicated by the additional roles of MR in glycoprotein uptake and immunity (Lee et al., 2002; Martinez-Pomares, 2012). A recent study used in vivo imaging to demonstrate MR-dependent uptake of injected labeled collagen by macrophages (Madsen et al., 2013).

MR and Endo180 share a common domain structure with two other receptors that are not believed to internalize collagen, phospholipase A_2 receptor (PLA_2R) and DEC-205 (East and Isacke, 2002). The large extracellular region of each of these receptors consists of an N-terminal ricin B-like domain, followed by a fibronectin type II (FN2) domain and eight (MR, Endo180, PLA_2R) or ten (DEC-205) C-type lectin (CTL) domains. The cytoplasmic regions are short and contain endocytosis motifs. FN2 domains in fibronectin and MMPs mediate gelatin binding (Banyai et al., 1994; Murphy et al., 1994; Pickford et al., 2001), and the same function has been demonstrated for the FN2 domains of MR (Napper et al., 2006) and

Table 1. Crystallographic Statistics of the Endo180 D1-4 Structure

Crystal Form	Monoclinic	Trigonal
Synchrotron beamline	Diamond I04-1	Diamond I04
Wavelength (Å)	0.92	0.98
Space group	C2	$P3_221$
Unit cell		
a, *b*, *c* (Å)	126.37, 92.37, 127.65	86.99, 86.99, 321.1
α, β, γ (°)	90, 100.31, 90	90, 90, 120
Solvent content (%)	62	59
Resolution range (Å)[a]	62.8–2.48 (2.54–2.48)	75.3–3.36 (3.45–3.36)
R_{merge}	0.049 (0.481)	0.181 (0.970)
Completeness (%)	97.8 (98.4)	99.4 (99.4)
Multiplicity	3.3 (3.4)	5.5 (5.6)
$<I/\sigma(I)>$	15.4 (2.2)	10.8 (2.3)
$CC_{1/2}$	0.994 (0.783)	0.989 (0.635)
Unique reflections	50,150	20,824
Protein atoms	6,960	6,833
Solvent atoms	163 H_2O, 4 Na^+, 4 SO_4^{2-}	
R_{work}	0.194 (0.282)	0.220 (0.320)
R_{free}	0.244 (0.314)	0.306 (0.409)
Rmsd bonds (Å)	0.006	0.005
Rmsd angles (°)	0.90	0.98
Ramachandran favored (%)[b]	95.8	93.4
Ramachandran outliers (%)[b]	0	0

[a]Values in parentheses are for the highest-resolution shell.
[b]Calculated with MolProbity (Chen et al., 2010).

Endo180 (Wienke et al., 2003). Why PLA_2R and DEC-205 do not bind and internalize collagen is not entirely clear. A study using chimeric proteins indicated that they lack critical determinants not only in FN2 but also in the neighboring domains (Jürgensen et al., 2014).

The ricin-like domain of MR binds sulfated carbohydrates (Fiete et al., 1998). The structural basis for this activity, which is not shared by Endo180 (East et al., 2002), has been revealed by X-ray crystallography (Liu et al., 2000). Of the eight CTL domains present in MR and Endo180, only CTL4 in MR and CTL2 in Endo180 appear to bind carbohydrates (East et al., 2002; Feinberg et al., 2000; Jürgensen et al., 2011). Endo180 binds Ca^{2+}-dependently to mannose, fucose, and *N*-acetylglucosamine, but not to galactose (East et al., 2002). This lectin activity has been shown to augment the binding of glycosylated collagens to Endo180 (Jürgensen et al., 2011).

Low-resolution structures of MR and Endo180 have been obtained by electron microscopy (Boskovic et al., 2006; Rivera-Calzada et al., 2003), but atomic structures of the collagen-binding regions have been lacking. Here, we present the crystal and solution structures of domains 1–4 (D1-4) of Endo180 and a mutational analysis of collagen binding. The FN2 domain is revealed to be integrated into an L-shaped structure that extends the collagen-binding trench of the FN2 domain into the neighboring CTL1 domain. The overall structure and collagen binding

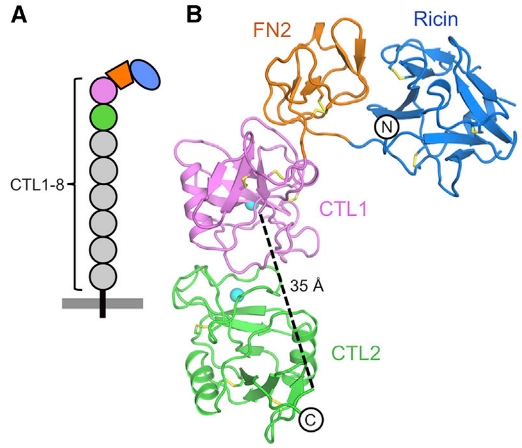

Figure 1. Crystal Structure of Endo180 D1-4

(A) Domain organization of Endo180. The plasma membrane is indicated by a gray bar and the crystallized D1-4 region is colored.

(B) Cartoon representation of the Endo180 D1-4 structure with the ricin-like domain shown in blue, the FN2 domain in orange, the CTL1 domain in pink, and the CTL2 domain in green. Disulfide bonds and sodium ions are shown as yellow sticks and cyan spheres, respectively. The N and C termini are labeled. The disordered CTL1-CTL2 linker (residues 364–378) is indicated by a broken line.

See also Figures S1 and S2.

are maintained at pH 5.5 and in the absence of Ca^{2+}, suggesting that ligand release in endosomes is not regulated by changes within the D1-4 region of Endo180.

RESULTS

Crystal Structure of Endo180 D1-4

We chose the D1-4 region of Endo180 for our crystallization trials, as it contains both the collagen- and carbohydrate-binding sites of this receptor (East et al., 2002; Jürgensen et al., 2011). We obtained two crystal forms of the natively glycosylated D1-4 region, one of which diffracted to 2.5 Å resolution and allowed the structure to be determined (Table 1). The overall structure of Endo180 D1-4 is the same in both crystal forms (the root-mean-square deviations [rmsd] between the four crystallographically independent copies range from 1.2 to 1.4 Å) and only the high-resolution structure is described in the following. The Endo180 D1-4 region adopts an L-shaped structure with a short arm (~60 Å length) consisting of the ricin-like and FN2 domains, and a longer arm (~80 Å length) consisting of the two CTL domains (Figure 1). The corner of the L is flattened and presents a contiguous FN2-CTL1 surface that includes FN2 residues predicted to be involved in collagen binding (Wienke et al., 2003). There is no electron density for residues 101–104 and 137–155 in the ricin-like domain and residues 364–378 in the CTL1-CTL2 linker. The relative arrangement of the two CTL domains shown in Figure 1B is the only plausible one given the limitations imposed by the length of the linker. Furthermore, the same overall structure is seen in two independent crystal forms and also in solution (see below). The inter-domain interfaces are sizable (ricin-FN2, 552 $Å^2$; FN2-CTL1, 674 $Å^2$; CTL1-CTL2, 662 $Å^2$), suggestive of a stable arrangement with limited inter-domain

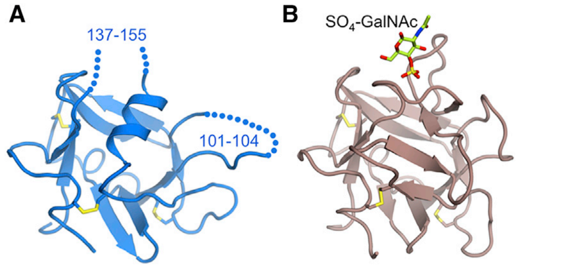

Figure 2. Comparison of the Ricin-like Domains of Endo180 and MR

The ricin-like domains of (A) Endo180 and (B) MR (Liu et al., 2000) are shown in the same orientation. The disordered loop regions in Endo180 are indicated by dotted lines. The 4-SO_4-GalNAc molecule bound to MR is shown in atomic detail.

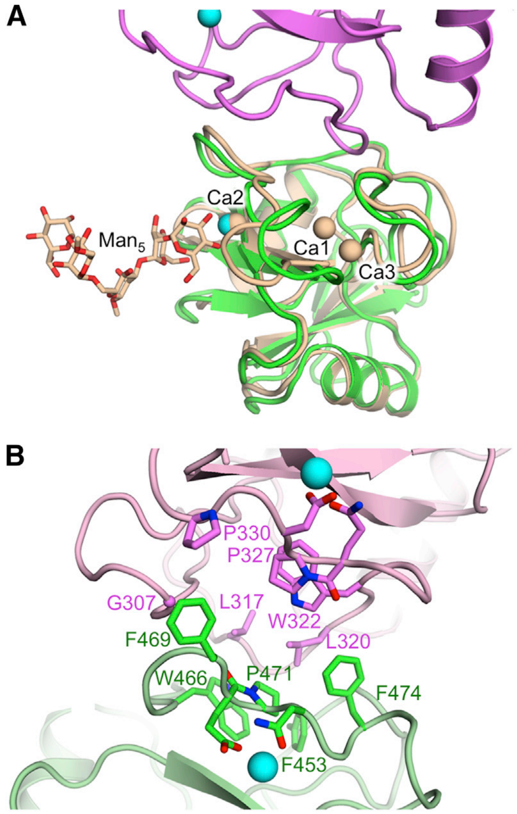

Figure 3. C-Type Lectin Domains 1 and 2 of Endo180

(A) Superposition of CTL2 of Endo180 (green) with rat mannose-binding protein bound to an oligomannose ligand (wheat) (Weis et al., 1992). The Endo180 CTL1 domain is shown in pink. Sodium and calcium ions are shown as cyan and wheat-colored spheres, respectively.

(B) The CTL1-CTL2 interface in Endo180. CTL1 and CTL2 are shown in pink and green, respectively. Selected residues are shown as sticks.

flexibility. The D1-4 region contains five predicted *N*-linked glycosylation sites. We observed weak electron density for the glycans at Asn69 and Asn497; the glycosylation sites at Asn102, Asn140, and Asn364 are located in the disordered loop regions mentioned above.

The ricin-like domain of Endo180 (residues 41–174) consists of eight antiparallel β strands arranged in two sheets. The six cysteines form three disulfide bonds: 54–68, 93–112, and 123–168. Compared with the regular β trefoil of the homologous domain in MR (Liu et al., 2000), the ricin-like domain of Endo180 lacks four β strands corresponding to one leaf of the trefoil (Figure 2). Their space is taken up by an orthogonal α helix, which contributes three critical leucines (Leu126, Leu130, and Leu134) to the hydrophobic core. The structural differences between MR and Endo180 in this region are intriguing given that the two domains share 29% sequence identity (Figure S1). The ricin-like domain of MR binds sulfated carbohydrates, and a co-crystal structure with 4-SO_3-GalNAc revealed that the galactose ring stacks against Trp117 (Liu et al., 2000). This residue is replaced by Ser155 in Endo180. MR residues interacting with the sulfate group are also not conserved in Endo180, explaining the lack of sulfated carbohydrate binding to Endo180 (East et al., 2002).

The FN2 domain, the most conserved domain among members of the C-type MR family, spans residues 175–231 and contains four cysteines forming two disulfide bonds: 187–213 and 201–228. The FN2 fold consists of two perpendicular β sheets, each made from two antiparallel strands, and a number of loops. These elements form a solvent-accessible depression rich in aromatic residues, which in other FN2-containing proteins have been demonstrated to be involved in gelatin binding (Figure S2) (Briknarova et al., 1999; Erat et al., 2013; Xu et al., 2009). Additional binding determinants are provided by the protruding loop connecting the two β sheets (Jürgensen et al., 2014).

The CTL domains 1 and 2 of Endo180 are very similar to each other (rmsd of 1.5 Å for 125 equivalent Cα atoms) and to other CTL domains (see below). The CTL fold is characterized by two three-stranded antiparallel β sheets (β1-β2-β6 and β3-β4-β5) with two flanking α helices inserted between β2 and β3 (Weis et al., 1998). Each Endo180 CTL domain contains the conserved pair of nested disulfide bonds (266–359 and 335–351 in CTL1, 410–504 and 481–496 in CTL2) and a third N-terminal disulfide bond (235–248 in CTL1 and 382–393 in CTL2). The ~40 residues between β3 and β4 are folded into elaborate loop structures that contain the canonical Ca^{2+}-binding sites of CTL domains (Weis et al., 1998). No Ca^{2+} ions were present in our crystallization solution, but we observed electron density for a metal ion (most likely Na^+) in each CTL domain in a position corresponding to the canonical Ca^{2+} "site 2" (Weis et al., 1998). Na^+ binding to a CTL domain has been observed previously (Feinberg et al., 2013). The Na^+ ions in Endo180 are coordinated by Gln326, Asp328, Glu333, and Asn348 in CTL1, and by Glu470, Asn472 and Asp493 in CTL2. Comparison with a typical Ca^{2+}-loaded CTL domain structure (Figure 3A) suggests that the Endo180 D1-4 crystal structure is unlikely to change much upon Ca^{2+} binding.

The CTL1 and CTL2 domains interact via their elaborate Ca^{2+}-binding loops. The substantial domain interface (662 $Å^2$) is dominated by hydrophobic residues, most prominently by Leu317, Leu320, and Trp322 from CTL1 and Phe453, Trp466, Pro471, and Phe474 from CTL2 (Figure 3B). These residues come from equivalent regions in the two CTL domains, and the CTL1-CTL2 arrangement indeed displays approximate two-fold symmetry.

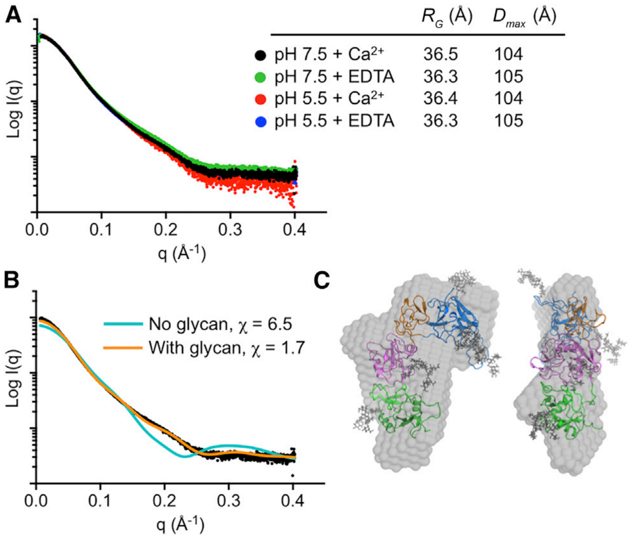

Figure 4. Small-Angle X-Ray Scattering Analysis of Endo180 D1-4

(A) Experimental data and derived R_G and D_{max} values at four different buffer conditions, as indicated. The pH 5.5 + EDTA data are obscured by the pH 7.5 + Ca^{2+} data.

(B) Fit of back-calculated curves to the pH 7.5 + Ca^{2+} data. The cyan and orange curves were computed, respectively, from the crystal structure and an all-atom model with added loops and glycans (Guttman et al., 2013).

(C) Best-fit model from (B) superimposed on the ab initio bead model (Franke and Svergun, 2009) calculated from the pH 7.5 + Ca^{2+} data. The two views are related by a 90° rotation about the vertical axis.

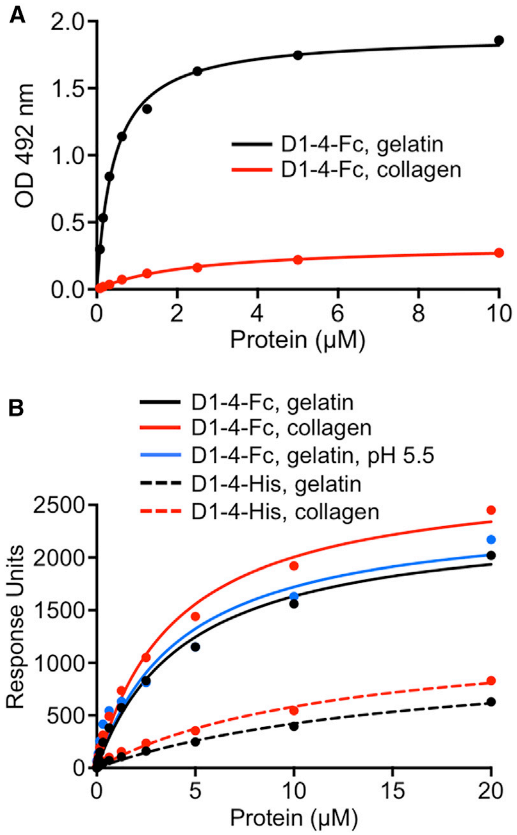

Figure 5. Collagen Binding by Endo180 D1-4

(A) Solid-phase assay of D1-4-Fc binding to immobilized type I collagen and gelatin (heat-denatured type I collagen). The data shown are representative of five independent experiments carried out in duplicate. For derived K_D values, see the text. Endo180 D1-4-Fc binding to an uncoated control surface is negligible.

(B) SPR analysis of D1-4-Fc (dimer) and D1-4-His (monomer) binding to immobilized type I collagen and gelatin. The data shown are representative of two independent experiments. For derived K_D values, see the text.

Unless indicated otherwise, the experiments were done at pH 7.5. See also Figure S3.

Solution Structure of Endo180 D1-4

Given that we did not observe electron density for the CTL1-CTL2 linker in the Endo180 D1-4 crystal structure, we wanted to confirm the domain arrangement in solution. Because pH-dependent conformational changes in the Endo180 D1-4 region were observed by electron microscopy (Boskovic et al., 2006), we performed small-angle X-ray scattering (SAXS) experiments with Endo180 D1-4 at pH 7.5 and 5.5, in the presence and absence of Ca^{2+}. The scattering curves obtained at these conditions were very similar, as were the derived R_G and D_{max} values (Figure 4A). A SAXS curve back-calculated from the crystal structure using FoXS (Schneidman-Duhovny et al., 2010) did not give a good fit to the experimental data, but an excellent fit was obtained once the missing loops and glycans were added to the model (Guttman et al., 2013) (Figure 4B). The resulting model of glycosylated Endo180 D1-4 agrees well with the low-resolution shape reconstructed ab initio from the SAXS data (Figure 4C). The SAXS experiments demonstrate that the Endo180 D1-4 crystal structure is representative of the solution structure at physiological pH and in the presence of Ca^{2+}. Moreover, they failed to detect any large conformational changes between pH 5.5 and pH 7.5.

Identification of the Collagen-Binding Site

Previous studies established that Endo180 binds not only to triple-helical collagens, but also to denatured collagen (gelatin) (Martinez-Pomares et al., 2006; Wienke et al., 2003). Endo180 binding to native or denatured type collagen I does not require Ca^{2+}, whereas there is a Ca^{2+}-dependent enhancement of binding to the more highly glycosylated type IV collagen (Jürgensen et al., 2011). We performed solid-phase binding experiments with immobilized type I collagen, with and without heat denaturation of the collagen prior to coating. In the solid-phase assay, a dimeric Fc-tagged Endo180 D1-4 construct bound to gelatin substantially better than to native collagen (gelatin: K_D = 0.42 ± 0.03 μM, B_{max} = 1.90 ± 0.03 optical density [OD]; collagen: K_D = 2.5 ± 0.25 μM, B_{max} = 0.34 ± 0.02 OD) (Figure 5A), and this behavior was the same in the presence or absence of Ca^{2+} (Figure S3). In surface plasmon resonance (SPR) experiments, the dimeric D1-4-Fc construct bound to gelatin and collagen with essentially identical K_D values of 4.5 ± 0.65 and 4.0 ± 0.60 μM, respectively, and a monomeric His-tagged construct also discriminated little between gelatin and collagen (K_D values of 17 ± 3.5 and 13 ± 2.4 μM, respectively) (Figure 5B). Thus, the parameters of the interaction are sensitive to the mode of ligand presentation, as might be expected for a complex ligand such as (denatured) collagen. The relatively weak collagen binding

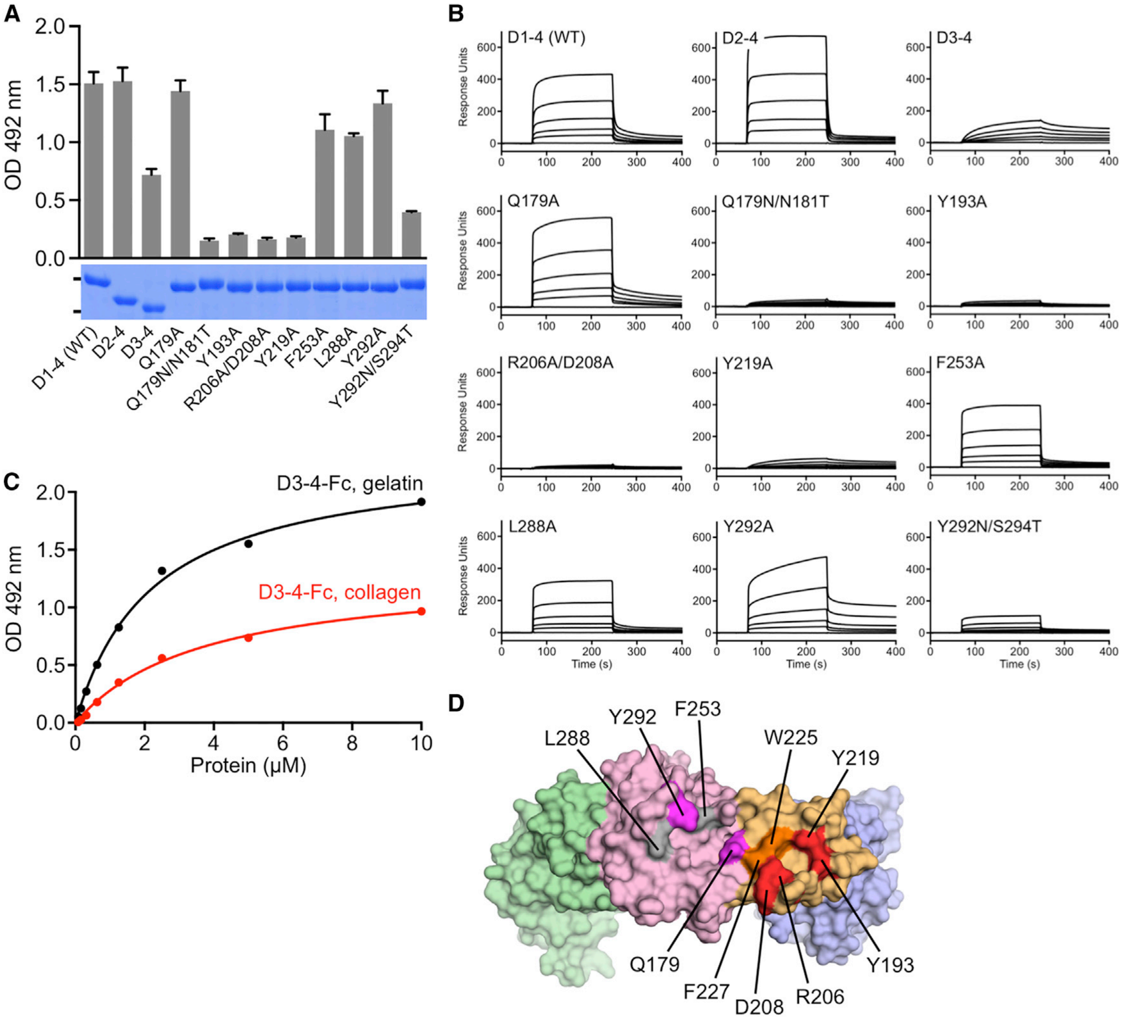

Figure 6. Mapping of the Collagen-Binding Site of Endo180 D1-4

(A) Solid-phase assay of Fc-tagged Endo180 proteins binding to immobilized gelatin. All proteins were tested at a fixed concentration of 1 μM. The data are mean ± SE (n = 3). The Coomassie blue-stained SDS-PAGE gel shows the proteins used in the assay, with the positions of the 100- and 60-kDa markers indicated on the left.

(B) SPR analysis of Fc-tagged Endo180 proteins binding to immobilized gelatin. Raw sensorgrams are shown for five concentrations (2, 1, 0.5, 0.25 and 0.125 μM) and a buffer injection.

(C) Solid-phase assay of Endo180 D3-4-Fc binding to immobilized type I collagen and gelatin. The K_D values derived from the fits are 3.7 ± 0.43 and 2.2 ± 0.15 μM, respectively. Endo180 D3-4-Fc binding to an uncoated control surface is negligible.

(D) Point mutations mapped onto the Endo180 D1-4 crystal structure (domains colored as in Figure 1B): red, mutation to alanine reduces collagen binding; magenta, mutation to an *N*-linked glycosylation site reduces collagen binding; gray, mutation to alanine has no effect on collagen binding; orange, mutation to alanine abolished protein secretion.

See also Figure S4.

observed in SPR is consistent with previous qualitative observations (Jürgensen et al., 2014; Martinez-Pomares et al., 2006; Napper et al., 2006; Wienke et al., 2003). Finally, we used SPR to show that Endo180 D1-4-Fc binds to gelatin equally well at neutral pH and at pH 5.5, a condition representing the acidic environment within endosomes (Figure 5B).

Previous studies showed that the D1-3 region of Endo180 contains all the binding determinants for type I collagen (Jürgensen et al., 2011). To assess the contributions of individual Endo180 domains to collagen/gelatin binding, we successively deleted domains from the N terminus. For these experiments we used Fc-tagged constructs because of their higher sensitivity, and to allow comparison between the two binding assays. Deletion of the ricin-like domain (D2-4-Fc construct) did not affect binding in the solid-phase assay (Figure 6A) or in SPR (Figure 6B). Deletion of both the ricin-like and FN2 domain (D3-4-Fc construct) reduced binding, but did not abolish it, indicating a contribution of CTL1 to collagen binding. Notably, the D3-4-Fc construct showed slower on and off rates in SPR compared with the step profiles observed with D1-4-Fc (Figure 6B), and it discriminated less between collagen and gelatin in the solid-phase assay (Figure 6C).

To identify collagen/gelatin-binding residues in the context of an intact Endo180 D1-4 structure, we generated 11 mutants (Figure 6D). Five mutations targeted residues in the FN2 domain whose counterparts in other FN2 domains are known to be important for gelatin binding (Briknarova et al., 1999; Mikhailova et al., 2012; Xu et al., 2009): Y193A, R206A/D208A, Y219A, W225A, and F227A (R206 and D208 were mutated together, as they form a salt bridge). The first three mutants were secreted by HEK293 cells, indicating correct folding. In support of this notion, mutation of residues analogous to Tyr193 and Arg206 did not perturb the FN2 fold in MMP-2 (Xu et al., 2009). Gln179 was targeted, as it is located within a loop of the Endo180 FN2 domain close to the interface with CTL1; this residue was mutated to Ala and to a sequon for *N*-linked glycosylation (Q179N/N181T). Finally, we mutated three hydrophobic residues in the CTL1 domain that are on the same face of the Endo180 structure as the presumed gelatin-binding site in the FN2 domain: F253A, L288A, and Y292A. We also introduced a sequon for glycan *N*-linked glycosylation at position 292 (Y292N/S294T). The engineered glycans at position 179 and 292 were indeed incorporated, as evidenced by a shift to higher mass on SDS-PAGE (Figure 6A).

Four Endo180 mutations essentially abolished binding to gelatin in the solid-phase assay: Y193A, R206A/D208A, Y219A, and Q179N/N181T (Figure 6A). They include FN2 residues suspected to be involved in binding (Tyr193, Arg206, Asp208, Tyr219), as well as a residue closer to the adjoining CTL1 domain, Gln179. Given that a bulky glycan at position 179 was required to abolish gelatin binding (the Q179A mutant did bind to gelatin), Gln179 is unlikely to make an essential direct contact with the collagen chain(s), unlike the aforementioned residues. Of the mutations in the CTL1 domain, only the engineered glycan at position 292 reduced gelatin binding. Very similar results were obtained with SPR, both for gelatin (Figure 6B) and native collagen (data not shown). From the collective data, we conclude that collagen/gelatin binds primarily to the canonical ligand-binding site of the FN2 domain, but that additional contacts are likely to be made with the extended FN2-CTL1 surface (Figures 6D and S4). The effect of the engineered glycan at position 292 could be due to sterical hindrance of collagen binding exclusively to the FN2 domain, but this interpretation would not explain the significant residual collagen binding of the D3-4-Fc construct, which lacks the FN2 domain.

DISCUSSION

Our crystal structure of the D1-4 region of Endo180 affords the first detailed view of the collagen-binding site of C-type MRs. The critical FN2 domain is revealed to be integrated into a seemingly rigid L-shaped structure that allows the adjacent CTL1 domain to participate in collagen binding. This finding is in agreement with a recent biochemical study, which showed that transfer of a functional FN2 domain from Endo180 was not sufficient to enable collagen internalization by the C-type MR family members PLA_2R and DEC-205 (Jürgensen et al., 2014). Presumably, differences within the putative collagen-binding trench in CTL1 (or within the FN2-CTL1 interface) account for the failure of the chimeric proteins to bind collagen. A comparison of FN2 domains shows that the critical collagen-binding residues of Endo180 are conserved in MR (Figure S1), consistent with the established role of MR in collagen uptake (Madsen et al., 2013). DEC-205 lacks critical collagen-binding determinants of Endo180 (Tyr193, Arg206, Asp208), but we can see no obvious reason why PLA_2R should not bind collagen. Indeed, collagen binding by the FN2-CTL1-CTL2 region of PLA_2R has been reported in another study (Takahashi et al., 2015). Ca^{2+}-dependent carbohydrate binding to the CTL2 domain augments binding of glycosylated type IV collagen to Endo180, whereas this effect was not observed with the less extensively glycosylated type I collagen (Jürgensen et al., 2011). Given the large distance between the binding sites for the collagen backbone and carbohydrates in Endo180, the enhanced binding of glycosylated collagen more likely results from cooperation of multiple receptors than from two-point binding by a single receptor.

MR and Endo180 are unique among FN2-containing proteins in that they bind not only gelatin but also triple-helical collagen. MMP-2 contains three FN2 domains that bind gelatin cooperatively with a K_D of 10 μM (Banyai et al., 1994). The gelatin-binding sites of the three FN2 domains (Briknarova et al., 1999; Xu et al., 2009) do not form a contiguous surface in the crystal structure of pro-MMP-2 (Morgunova et al., 1999), suggesting that a cooperative interaction is only possible with the more flexible single chains of gelatin and not with the rigid triple helix of native collagen. The gelatin-binding region of fibronectin consists of two FN2 domains flanked by fibronectin type I (FN1) domains, ^{6}FN1-^{1}FN2-^{2}FN2-^{8}FN1-^{9}FN1. Structural and biophysical studies with collagen-derived peptides have shown that a single collagen chain forms an antiparallel β strand on the outside of the ^{8}FN1-^{9}FN1 pair (Erat et al., 2009) and may simultaneously contact the canonical gelatin-binding sites of the ^{1}FN2-^{2}FN2 pair (Erat et al., 2013). As in MMP-2, the ligand-binding mode is not compatible with a triple-helical collagen structure. In contrast, the collagen-binding trench spanning the FN2 and CTL1 domains in Endo180 seems uniquely suited for accommodating triple helices as well as single chains.

The binding site(s) for Endo180 in collagen are unknown. Given that Endo180 appears to function as a clearance receptor for cleaved and denatured collagen (see Introduction), it intuitively seems more likely that it recognizes some generic feature of a collagen-derived polypeptide rather than a specific sequence. Indeed, binding studies provide some evidence for multiple Endo180-binding sites in type I collagen. In our SPR experiments with immobilized collagen, we observed relatively weak Endo180 binding with fast on and off rates, likely representing a monovalent interaction with single sites. In contrast, when Endo180 is immobilized at high density and collagen is flowed over this surface (Jürgensen et al., 2011; Madsen et al., 2007), the interaction is characterized by slower kinetics and apparent affinities in the nanomolar range, likely representing a multivalent interaction of a single triple helix with several receptors. Libraries of triple-helical collagen-derived peptides have been invaluable in defining binding sites for other collagen receptors, e.g. the discoidin domain receptors (Carafoli et al., 2009; Konitsiotis et al., 2008). Interrogating such libraries with Endo180 may not identify the physiologically relevant binding sites in denatured collagen, however.

Previous single-particle reconstructions of negatively stained Endo180 concluded that the ricin-like domain interacts with

CTL2 at neutral pH, and that this interaction is broken at pH 5.4 (Boskovic et al., 2006; Rivera-Calzada et al., 2003). This interpretation is not consistent with our crystal structure and solution SAXS data, which reveal a more open structure that does not substantially change with pH. A recent cryoelectron microscopy structure of DEC-205 at pH 6 revealed an interaction of the ricin-like domain with CTL3 (Cao et al., 2015). Inspection of the Endo180 density obtained from electron microscopy (Rivera-Calzada et al., 2003) suggests that the domains may have been incorrectly assigned and that it is CTL3 which interacts with the ricin-like domain also in Endo180. A single-particle reconstruction of MR revealed a globular head and an S-shaped tail, but the resolution was not sufficient to unambiguously assign the D1-4 region within the head (Boskovic et al., 2006). A more extended MR structure was inferred from analytical ultracentrifugation experiments (Napper et al., 2001). DEC-205 and the avian MR homolog FcRU adopt compact structures at pH 6 and become more extended at higher pH (Cao et al., 2015; He and Bjorkman, 2011). Conformational changes thus appear to be a recurring theme in the C-type MR family. Although we cannot exclude that the inter-domain interfaces in our Endo180 D1-4 structure might be broken under certain conditions, we think it more likely that the D1-4 region behaves as a rigid collagen-binding module in MR and Endo180, with the remaining six CTL domains providing the flexibility for any large-scale structural changes. Limited trypsin digestion of MR indicated stable association of CTL1-2, CTL4-5, and CTL7-8 (Napper et al., 2001). It is tempting to speculate that these stable pairs resemble the head-to-head arrangement of CTL1-2 in our Endo180 crystal structure and that the pairs are linked more flexibly by the intervening domains, CTL3 and CTL6.

Structural changes in the MR and Endo180 ectodomains may be relevant for the mechanism of collagen release within endosomes, which is unknown. Endocytosed receptors that bind ligand tightly, such as epidermal growth factor receptor, are often degraded together with the ligand (Goh and Sorkin, 2013). In contrast, recycling receptors, such as MR and Endo180, must release their ligand before returning to the plasma membrane. Commonly the release is accomplished by mechanisms that disfavor ligand binding at the acidic pH and low Ca^{2+} concentration of late endosomes (Andersen and Moestrup, 2014). For instance, the low-density lipoprotein receptor structure at low pH revealed an autoinhibited conformation in which the N-terminal ligand-binding region is folded back against the membrane-proximal domains (Rudenko et al., 2002). Collagen binding to MR and Endo180 is not Ca^{2+}-dependent (Jürgensen et al., 2011; Martinez-Pomares et al., 2006), and we found that collagen binding to Endo180 D1-4 is not diminished at pH 5.5. Therefore, if there is pH-dependent regulation of ligand binding to Endo180, it would have to involve regions outside of D1-4. How collagen is released from internalized Endo180 is an important question for further study.

EXPERIMENTAL PROCEDURES

Expression Vectors

Coding sequences were amplified from a full-length cDNA of human Endo180 (a kind gift from Dr. Clare Isacke, Institute of Cancer Research, London, UK) and cloned into modified pCEP-Pu vectors (Kohfeldt et al., 1997). All pCEP-encoded proteins contain a vector-derived APLA sequence at the N terminus, and are either untagged or have hexahistidine or immunoglobulin G2 (IgG2) Fc tags at the C terminus (Hussain et al., 2006). The D1-4 construct comprises Endo180 residues 35–513 (UniProt: Q9UBG0), the D2-4 construct comprises residues 172–513, and the D3-4 construct comprises residues 231–513. Point mutations were introduced with strand-overlap extension PCR. All expression vectors were verified by DNA sequencing.

Protein Production

Human embryonic kidney HEK293 c18 cells (American Type Culture Collection) were used for protein production. The cells were grown at 37°C with 5% CO_2 in DMEM/F12 (Invitrogen) containing 10% fetal bovine serum, 2 mM glutamine, 10 U/ml penicillin, 100 μg/ml streptomycin, and 250 μg/ml geneticin. Cells were transfected with the expression vectors using Fugene (Roche Diagnostics) and selected with 1 μg/ml puromycin (Sigma). Transfected cells were grown to confluence in HYPERFlasks (Corning) or 525-cm^2 three-layer flasks (BD Biosciences), washed twice with PBS, and incubated with serum-free medium for up to 3 weeks with two medium exchanges per week.

For purification of His-tagged Endo180 D1-4, the filtered serum-free cell culture supernatant was adjusted to a final concentration of 20 mM Na-HEPES (pH 7.5) and loaded onto a 5-ml HisTrap Excel column (GE Healthcare) using an Äkta Purifier (GE Healthcare). The column was washed with PBS and the protein was eluted with PBS containing 500 mM imidazole. Fractions containing protein were concentrated using a Vivaspin centrifugal device (Sartorius) and further purified on a Superdex 200 10/300 Gl column (GE Healthcare) using 20 mM Tris-HCl (pH 7.5) and 150 mM NaCl as the running buffer.

The Fc-tagged Endo180 proteins were purified using 1-ml protein A HP columns (GE Healthcare). The columns were washed with PBS and the proteins were eluted with 100 mM citrate buffer (pH 3.0). The 1-ml fractions were immediately neutralized by mixing with 100 μl of 1 M Tris-HCl (pH 8.5) and dialyzed against PBS.

For purification of untagged Endo180 D1-4, the serum-free cell culture supernatant was exchanged into 50 mM Na-HEPES (pH 7.5) using a VIVAFLOW 200 cross-filtration device (Sartorius Stedim Biotech). The concentrated solution was loaded onto a HiTrap DEAE FF column (5 ml, GE Healthcare) and protein eluted in a stepwise fashion with 50 mM Na-HEPES (pH 7.5) containing 100, 150, 200, 250, and 500 mM NaCl. The fractions eluted by 150 mM NaCl were combined and further purified on a Superdex 200 10/300 Gl column (GE Healthcare) using 20 mM HEPES (pH 7.5), 150 mM NaCl, and 10 mM EDTA as the running buffer.

Crystallization

Screening was done at room temperature by the sitting-drop vapor diffusion method using 96-well plates (Greiner) and a range of commercial screens (Hampton Research, Molecular Dimensions). A Mosquito nanoliter robot (TTP Labtech) was used to set up 200-nl sitting drops. His-tagged Endo180 D1-4 crystallized under a wide range of conditions, but none of the crystals diffracted to high resolution. The best crystals that could be obtained were grown from an 8-mg/ml protein solution (in 20 mM Tris-HCl [pH 7.5], 150 mM NaCl) using 100 mM BIS-TRIS-HCl (pH 6.5) and 18% polyethylene glycol monomethyl ether 5000 as precipitant. The crystals were harvested in reservoir solution supplemented with 25% glycerol and flash-frozen in liquid nitrogen. Crystals of untagged Endo180 D1-4 were grown from a 10-mg/ml protein solution (in 20 mM HEPES [pH 7.5], 150 mM NaCl, 10 mM EDTA) using condition H5 of the Morpheus screen (Molecular Dimensions), which contains a mixture of amino acids and polyethylene glycols buffered at pH 7.5. These crystals were flash-frozen directly from the sitting drops.

Structure Determination

Diffraction data were collected at 100 K at beamlines IO4 and IO4-1 of the Diamond Light Source, Oxfordshire, UK. The data were indexed, integrated, scaled, and merged with XIA2 (Winter, 2010). The crystals of His-tagged and untagged Endo180 D1-4 were found to belong to space groups $P3_221$ and C2, respectively, and each to contain two copies of Endo180 D1-4 in the asymmetric unit. Both crystal forms exhibited anisotropic diffraction limits (trigonal crystals: <3.3 Å along a* and b*, ~4.0 Å along c*; monoclinic crystals: <2.5 Å along b* and c*, ~3.0 Å along a*).

The monoclinic Endo180 D1-4 structure was solved by molecular replacement using the program PHASER (McCoy et al., 2007). Using the CTL of aggrecan (PDB: 1TDQ) as a search model, four solutions were found, corresponding to CTL1 and CTL2 of the two D1-4 copies in the asymmetric unit. The CTLs were fixed and the FN2 domains placed using the first FN2 domain of fibronectin (PDB: 3M7P) as a search model. Finally, the ricin-like domains were placed using the ricin-like domain of MR (PDB: 1DQG) as a search model. Manual rebuilding and refinement were done using Coot (Emsley and Cowtan, 2004) and PHENIX (Adams et al., 2010). The refined monoclinic Endo180 D1-4 structure was used to solve the trigonal crystal form. Figures were generated using PyMOL (http://www.pymol.org/).

Small-Angle X-Ray Scattering

SAXS data were collected at beamline B21 of the Diamond Light Source over a momentum transfer range of 0.028 $Å^{-1} < q < 0.403$ $Å^{-1}$ from untagged Endo180 D1-4 in four different buffers: 20 mM Na-HEPES (pH 7.5), 150 mM NaCl with either 10 mM EDTA or 5 mM $CaCl_2$; 20 mM Na-MES (2-(*N*-morpholino)ethanesulfonic acid) (pH 5.5), 150 mM NaCl with either 10 mM EDTA or 5 mM $CaCl_2$. Data were collected from three sequential 2-fold dilutions, starting at the following concentrations: 17 mg/ml (pH 7.5 + EDTA), 10 mg/ml (pH 7.5 + Ca^{2+}), 13 mg/ml (pH 5.5 + EDTA), and 7 mg/ml (pH 5.5 + Ca^{2+}). The data were analyzed, buffer-subtracted, scaled, and merged using the Scatter software package (Forster et al., 2010). R_G and D_{max} values are given in Figure 4A. Shape estimation was carried out with DAMMIF/DAMMIN (Franke and Svergun, 2009), as implemented in Scatter. In brief, 20 ab initio models were generated from each dataset, which were then superimposed and averaged using DAMAVER. The mean normalized spatial discrepancy values were 0.71 (pH 7.5 + EDTA), 0.68 (pH 7.5 + Ca^{2+}), 0.71 (pH 5.5 + EDTA), and 0.59 (pH 5.5 + Ca^{2+}). The averaged models from DAMAVER were refined to convergence using DAMMIN, giving final χ values of 0.269 (pH 7.5 + EDTA), 0.372 (pH 7.5 + Ca^{2+}), 0.309 (pH 5.5 + EDTA), and 0.231 (pH 5.5 + Ca^{2+}). Comparison with the crystal structure was carried out as described by Guttman et al. (2013).

Solid-Phase Binding Assay

The solid-phase binding assay was performed as described by Leitinger (2003). Denatured collagen (gelatin) was prepared by heating a 1-mg/ml solution of rat-tail type I collagen (Sigma) in 100 mM acetic acid to 67°C for 30 min. Native collagen and gelatin were coated onto flat-bottomed 96-well plates (Nunc MaxiSorp) at a concentration of 10 μg/ml in 100 mM Tris-HCl (pH 8.5) and 150 mM NaCl. Unreacted surfaces were blocked with PBS containing 0.1 mg/ml bovine milk κ-casein (Sigma) and 0.05% Tween 20. For the experiment shown in Figure S3, PBS was replaced by 20 mM Na-HEPES (pH 7.5) and 150 mM NaCl containing either 10 mM EDTA or calcium chloride. Fc-tagged Endo180 proteins diluted in blocking buffer were added for 2 hr. After washing, bound Endo180 proteins were detected by antihuman IgG antibody conjugated to horseradish peroxidase (Sigma) and SIGMAFAST OPD solution (Sigma). The plates were measured at 492 nm using a Tecan Sunrise plate reader. The data were fitted to a single-site binding model using GraphPad Prism.

Surface Plasmon Resonance

The experiments were performed with a Biacore 3000 instrument (GE Healthcare), and collagen and gelatin covalently attached to a CM5 sensor chip (GE Healthcare). A 10-μg/ml solution of collagen in 10 mM citrate buffer (pH 3.2) (either untreated or heat-denatured as described above) was flowed over the activated sensor chip surface until a response of ~1,500 resonance units (RU) was reached, and the remaining activated sites were then blocked using 1 M ethanolamine (pH 7.0). A reference surface was prepared in the same way without collagen.

The experiments were performed in 20 mM Na-HEPES (pH 7.5), 130 mM NaCl, 5 mM EDTA, 0.005% Tween 20, or with 20 mM Na-MES (pH 5.5) replacing the HEPES buffer. Endo180 proteins were passed over the sensor chip surface at a flow rate of 30 μl/min at concentrations ranging from 0 to 20 μM for wild-type D1-4 proteins and from 0 to 2 μM for all other proteins. After each injection, the surfaces were regenerated using a brief pulse of 2 M guanidine-HCl (pH 7.0). To confirm that this treatment did not denature the collagen surface, we measured binding of a recombinant SPARC protein that recognizes only triple-helical type I collagen (Giudici et al., 2008; Hohenester et al., 2008). Steady-state RU values were obtained using BIAevaluation software (GE Healthcare). The data were fitted to a single-site binding model using GraphPad Prism.

ACCESSION NUMBERS

The coordinates of the monoclinic and trigonal Endo180 D1-4 crystal structures have been deposited in PDB (PDB: 5AO5 and 5AO6, respectively).

SUPPLEMENTAL INFORMATION

Supplemental Information includes four figures and can be found with this article online at http://dx.doi.org/10.1016/j.str.2015.09.004.

AUTHOR CONTRIBUTIONS

All authors contributed to experimental design and data analysis. P.P. made most of the proteins used in the study, determined the monoclinic crystal structure, and performed the solid-phase binding experiments. D.C.B. determined the trigonal crystal structure and performed the SAXS and SPR experiments. F.C. grew the trigonal crystals. T.L. and P.P. grew the monoclinic crystals. P.P., D.C.B., and E.H. wrote the manuscript.

ACKNOWLEDGMENTS

We thank Ezequiel Miron Sardiello, Nicola Coker Gordon, Chaitra Sathyaprakash, Ying Ma, Grace Chan, and Sanja Brolih for preliminary experiments; Katsuaki Inoue and James Doutch for help with SAXS experiments; and Clare Isacke, Maureen Taylor, and Kurt Drickamer for helpful discussions. We acknowledge Diamond Light Source for time on beamlines IO4 and IO4-1 under proposal MX9424, and for time on beamline B21 under proposal SM12060. The research was funded by a Wellcome Trust Senior Investigator Award to E.H. (ref. 101748/Z/13/Z).

Received: August 3, 2015
Revised: September 9, 2015
Accepted: September 14, 2015
Published: October 15, 2015

REFERENCES

Adams, P.D., Afonine, P.V., Bunkoczi, G., Chen, V.B., Davis, I.W., Echols, N., Headd, J.J., Hung, L.W., Kapral, G.J., Grosse-Kunstleve, R.W., et al. (2010). PHENIX: a comprehensive Python-based system for macromolecular structure solution. Acta Crystallogr. D Biol. Crystallogr. *66*, 213–221.

Andersen, C.B., and Moestrup, S.K. (2014). How calcium makes endocytic receptors attractive. Trends Biochem. Sci. *39*, 82–90.

Banyai, L., Tordai, H., and Patthy, L. (1994). The gelatin-binding site of human 72 kDa type IV collagenase (gelatinase A). Biochem. J. *298*, 403–407.

Bonnans, C., Chou, J., and Werb, Z. (2014). Remodelling the extracellular matrix in development and disease. Nat. Rev. Mol. Cell Biol. *15*, 786–801.

Boskovic, J., Arnold, J.N., Stilion, R., Gordon, S., Sim, R.B., Rivera-Calzada, A., Wienke, D., Isacke, C.M., Martinez-Pomares, L., and Llorca, O. (2006). Structural model for the mannose receptor family uncovered by electron microscopy of Endo180 and the mannose receptor. J. Biol. Chem. *281*, 8780–8787.

Briknarova, K., Grishaev, A., Banyai, L., Tordai, H., Patthy, L., and Llinas, M. (1999). The second type II module from human matrix metalloproteinase 2: structure, function and dynamics. Structure *7*, 1235–1245.

Cao, L., Shi, X., Chang, H., Zhang, Q., and He, Y. (2015). pH-dependent recognition of apoptotic and necrotic cells by the human dendritic cell receptor DEC205. Proc. Natl. Acad. Sci. USA *112*, 7237–7242.

Carafoli, F., Bihan, D., Stathopoulos, S., Konitsiotis, A.D., Kvansakul, M., Farndale, R.W., Leitinger, B., and Hohenester, E. (2009). Crystallographic insight into collagen recognition by discoidin domain receptor 2. Structure *17*, 1573–1581.

Chen, V.B., Arendall, W.B., 3rd, Headd, J.J., Keedy, D.A., Immormino, R.M., Kapral, G.J., Murray, L.W., Richardson, J.S., and Richardson, D.C. (2010). MolProbity: all-atom structure validation for macromolecular crystallography. Acta Crystallogr. D Biol. Crystallogr. *66*, 12–21.

Curino, A.C., Engelholm, L.H., Yamada, S.S., Holmbeck, K., Lund, L.R., Molinolo, A.A., Behrendt, N., Nielsen, B.S., and Bugge, T.H. (2005). Intracellular collagen degradation mediated by uPARAP/Endo180 is a major pathway of extracellular matrix turnover during malignancy. J. Cell Biol. *169*, 977–985.

Danielsen, C.C. (1987). Thermal stability of human-fibroblast-collagenase-cleavage products of type-I and type-III collagens. Biochem. J. *247*, 725–729.

East, L., and Isacke, C.M. (2002). The mannose receptor family. Biochim. Biophys. Acta *1572*, 364–386.

East, L., Rushton, S., Taylor, M.E., and Isacke, C.M. (2002). Characterization of sugar binding by the mannose receptor family member, Endo180. J. Biol. Chem. *277*, 50469–50475.

East, L., McCarthy, A., Wienke, D., Sturge, J., Ashworth, A., and Isacke, C.M. (2003). A targeted deletion in the endocytic receptor gene Endo180 results in a defect in collagen uptake. EMBO Rep. *4*, 710–716.

Emsley, P., and Cowtan, K. (2004). Coot: model-building tools for molecular graphics. Acta Crystallogr. D Biol. Crystallogr. *60*, 2126–2132.

Engelholm, L.H., List, K., Netzel-Arnett, S., Cukierman, E., Mitola, D.J., Aaronson, H., Kjoller, L., Larsen, J.K., Yamada, K.M., Strickland, D.K., et al. (2003). uPARAP/Endo180 is essential for cellular uptake of collagen and promotes fibroblast collagen adhesion. J. Cell Biol. *160*, 1009–1015.

Erat, M.C., Slatter, D.A., Lowe, E.D., Millard, C.J., Farndale, R.W., Campbell, I.D., and Vakonakis, I. (2009). Identification and structural analysis of type I collagen sites in complex with fibronectin fragments. Proc. Natl. Acad. Sci. USA *106*, 4195–4200.

Erat, M.C., Sladek, B., Campbell, I.D., and Vakonakis, I. (2013). Structural analysis of collagen type I interactions with human fibronectin reveals a cooperative binding mode. J. Biol. Chem. *288*, 17441–17450.

Everts, V., van der Zee, E., Creemers, L., and Beertsen, W. (1996). Phagocytosis and intracellular digestion of collagen, its role in turnover and remodelling. Histochem. J. *28*, 229–245.

Feinberg, H., Park-Snyder, S., Kolatkar, A.R., Heise, C.T., Taylor, M.E., and Weis, W.I. (2000). Structure of a C-type carbohydrate recognition domain from the macrophage mannose receptor. J. Biol. Chem. *275*, 21539–21548.

Feinberg, H., Jegouzo, S.A., Rowntree, T.J., Guan, Y., Brash, M.A., Taylor, M.E., Weis, W.I., and Drickamer, K. (2013). Mechanism for recognition of an unusual mycobacterial glycolipid by the macrophage receptor mincle. J. Biol. Chem. *288*, 28457–28465.

Fields, G.B. (2013). Interstitial collagen catabolism. J. Biol. Chem. *288*, 8785–8793.

Fiete, D.J., Beranek, M.C., and Baenziger, J.U. (1998). A cysteine-rich domain of the "mannose" receptor mediates GalNAc-4-SO_4 binding. Proc. Natl. Acad. Sci. USA *95*, 2089–2093.

Forster, S., Apostol, L., and Bras, W. (2010). Scatter: software for the analysis of nano- and mesoscale small-angle scattering. J. Appl. Crystallogr. *43*, 639–646.

Franke, D., and Svergun, D.I. (2009). DAMMIF, a program for rapid ab-initio shape determination in small-angle scattering. J. Appl. Crystallogr. *42*, 342–346.

Giudici, C., Raynal, N., Wiedemann, H., Cabral, W.A., Marini, J.C., Timpl, R., Bächinger, H.P., Farndale, R.W., Sasaki, T., and Tenni, R. (2008). Mapping of SPARC/BM-40/osteonectin-binding sites on fibrillar collagens. J. Biol. Chem. *283*, 19551–19560.

Goh, L.K., and Sorkin, A. (2013). Endocytosis of receptor tyrosine kinases. Cold Spring Harb. Perspect. Biol. *5*, a017459.

Gross, J., and Nagai, Y. (1965). Specific degradation of the collagen molecule by tadpole collagenolytic enzyme. Proc. Natl. Acad. Sci. USA *54*, 1197–1204.

Guttman, M., Weinkam, P., Sali, A., and Lee, K.K. (2013). All-atom ensemble modeling to analyze small-angle x-ray scattering of glycosylated proteins. Structure *21*, 321–331.

He, Y., and Bjorkman, P.J. (2011). Structure of FcRY, an avian immunoglobulin receptor related to mammalian mannose receptors, and its complex with IgY. Proc. Natl. Acad. Sci. USA *108*, 12431–12436.

Hohenester, E., Sasaki, T., Giudici, C., Farndale, R.W., and Bächinger, H.P. (2008). Structural basis of sequence-specific collagen recognition by SPARC. Proc. Natl. Acad. Sci. USA *105*, 18273–18277.

Hussain, S.A., Piper, M., Fukuhara, N., Strochlic, L., Cho, G., Howitt, J.A., Ahmed, Y., Powell, A.K., Turnbull, J.E., Holt, C.E., et al. (2006). A molecular mechanism for the heparan sulfate dependence of Slit-Robo signaling. J. Biol. Chem. *281*, 39693–39698.

Jürgensen, H.J., Madsen, D.H., Ingvarsen, S., Melander, M.C., Gardsvoll, H., Patthy, L., Engelholm, L.H., and Behrendt, N. (2011). A novel functional role of collagen glycosylation: interaction with the endocytic collagen receptor uparap/ENDO180. J. Biol. Chem. *286*, 32736–32748.

Jürgensen, H.J., Johansson, K., Madsen, D.H., Porse, A., Melander, M.C., Sorensen, K.R., Nielsen, C., Bugge, T.H., Behrendt, N., and Engelholm, L.H. (2014). Complex determinants in specific members of the mannose receptor family govern collagen endocytosis. J. Biol. Chem. *289*, 7935–7947.

Kadler, K.E., Baldock, C., Bella, J., and Boot-Handford, R.P. (2007). Collagens at a glance. J. Cell Sci. *120*, 1955–1958.

Kohfeldt, E., Maurer, P., Vannahme, C., and Timpl, R. (1997). Properties of the extracellular calcium binding module of the proteoglycan testican. FEBS Lett. *414*, 557–561.

Konitsiotis, A.D., Raynal, N., Bihan, D., Hohenester, E., Farndale, R.W., and Leitinger, B. (2008). Characterization of high affinity binding motifs for the discoidin domain receptor DDR2 in collagen. J. Biol. Chem. *283*, 6861–6868.

Lee, S.J., Evers, S., Roeder, D., Parlow, A.F., Risteli, J., Risteli, L., Lee, Y.C., Feizi, T., Langen, H., and Nussenzweig, M.C. (2002). Mannose receptor-mediated regulation of serum glycoprotein homeostasis. Science *295*, 1898–1901.

Leitinger, B. (2003). Molecular analysis of collagen binding by the human discoidin domain receptors, DDR1 and DDR2. Identification of collagen binding sites in DDR2. J. Biol. Chem. *278*, 16761–16769.

Liu, Y., Chirino, A.J., Misulovin, Z., Leteux, C., Feizi, T., Nussenzweig, M.C., and Bjorkman, P.J. (2000). Crystal structure of the cysteine-rich domain of mannose receptor complexed with a sulfated carbohydrate ligand. J. Exp. Med. *191*, 1105–1116.

Madsen, D.H., Engelholm, L.H., Ingvarsen, S., Hillig, T., Wagenaar-Miller, R.A., Kjoller, L., Gardsvoll, H., Hoyer-Hansen, G., Holmbeck, K., Bugge, T.H., et al. (2007). Extracellular collagenases and the endocytic receptor, urokinase plasminogen activator receptor-associated protein/Endo180, cooperate in fibroblast-mediated collagen degradation. J. Biol. Chem. *282*, 27037–27045.

Madsen, D.H., Ingvarsen, S., Jürgensen, H.J., Melander, M.C., Kjoller, L., Moyer, A., Honore, C., Madsen, C.A., Garred, P., Burgdorf, S., et al. (2011). The non-phagocytic route of collagen uptake: a distinct degradation pathway. J. Biol. Chem. *286*, 26996–27010.

Madsen, D.H., Leonard, D., Masedunskas, A., Moyer, A., Jürgensen, H.J., Peters, D.E., Amornphimoltham, P., Selvaraj, A., Yamada, S.S., Brenner, D.A., et al. (2013). M2-like macrophages are responsible for collagen degradation through a mannose receptor-mediated pathway. J. Cell Biol. *202*, 951–966.

Martinez-Pomares, L. (2012). The mannose receptor. J. Leukoc. Biol. *92*, 1177–1186.

Martinez-Pomares, L., Wienke, D., Stillion, R., McKenzie, E.J., Arnold, J.N., Harris, J., McGreal, E., Sim, R.B., Isacke, C.M., and Gordon, S. (2006). Carbohydrate-independent recognition of collagens by the macrophage mannose receptor. Eur. J. Immunol. *36*, 1074–1082.

McCoy, A.J., Grosse-Kunstleve, R.W., Adams, P.D., Winn, M.D., Storoni, L.C., and Read, R.J. (2007). Phaser crystallographic software. J. Appl. Crystallogr. *40*, 658–674.

Mikhailova, M., Xu, X., Robichaud, T.K., Pal, S., Fields, G.B., and Steffensen, B. (2012). Identification of collagen binding domain residues that govern catalytic activities of matrix metalloproteinase-2 (MMP-2). Matrix Biol. *31*, 380–388.

Morgunova, E., Tuuttila, A., Bergmann, U., Isupov, M., Lindqvist, Y., Schneider, G., and Tryggvason, K. (1999). Structure of human pro-matrix metalloproteinase-2: activation mechanism revealed. Science *284*, 1667–1670.

Murphy, G., Nguyen, Q., Cockett, M.I., Atkinson, S.J., Allan, J.A., Knight, C.G., Willenbrock, F., and Docherty, A.J. (1994). Assessment of the role of the fibronectin-like domain of gelatinase A by analysis of a deletion mutant. J. Biol. Chem. *269*, 6632–6636.

Napper, C.E., Dyson, M.H., and Taylor, M.E. (2001). An extended conformation of the macrophage mannose receptor. J. Biol. Chem. *276*, 14759–14766.

Napper, C.E., Drickamer, K., and Taylor, M.E. (2006). Collagen binding by the mannose receptor mediated through the fibronectin type II domain. Biochem. J. *395*, 579–586.

Pickford, A.R., Smith, S.P., Staunton, D., Boyd, J., and Campbell, I.D. (2001). The hairpin structure of the $^{6}F1^{1}F2^{2}F2$ fragment from human fibronectin enhances gelatin binding. EMBO J. *20*, 1519–1529.

Rivera-Calzada, A., Robertson, D., MacFadyen, J.R., Boskovic, J., Isacke, C.M., and Llorca, O. (2003). Three-dimensional interplay among the ligand-binding domains of the urokinase-plasminogen-activator-receptor-associated protein, Endo180. EMBO Rep. *4*, 807–812.

Rudenko, G., Henry, L., Henderson, K., Ichtchenko, K., Brown, M.S., Goldstein, J.L., and Deisenhofer, J. (2002). Structure of the LDL receptor extracellular domain at endosomal pH. Science *298*, 2353–2358.

Schneidman-Duhovny, D., Hammel, M., and Sali, A. (2010). FoXS: a web server for rapid computation and fitting of SAXS profiles. Nucleic Acids Res. *38*, W540–W544.

Takahashi, S., Watanabe, K., Watanabe, Y., Fujioka, D., Nakamura, T., Nakamura, K., Obata, J.E., and Kugiyama, K. (2015). C-type lectin-like domain and fibronectin-like type II domain of phospholipase A_2 receptor 1 modulate binding and migratory responses to collagen. FEBS Lett. *589*, 829–835.

Weis, W.I., Drickamer, K., and Hendrickson, W.A. (1992). Structure of a C-type mannose-binding protein complexed with an oligosaccharide. Nature *360*, 127–134.

Weis, W.I., Taylor, M.E., and Drickamer, K. (1998). The C-type lectin superfamily in the immune system. Immunol. Rev. *163*, 19–34.

Wienke, D., MacFadyen, J.R., and Isacke, C.M. (2003). Identification and characterization of the endocytic transmembrane glycoprotein Endo180 as a novel collagen receptor. Mol. Biol. Cell *14*, 3592–3604.

Winter, G. (2010). xia2: an expert system for macromolecular crystallography data reduction. J. Appl. Crystallogr. *43*, 186–190.

Xu, X., Mikhailova, M., Ilangovan, U., Chen, Z., Yu, A., Pal, S., Hinck, A.P., and Steffensen, B. (2009). Nuclear magnetic resonance mapping and functional confirmation of the collagen binding sites of matrix metalloproteinase-2. Biochemistry *48*, 5822–5831.

Structure, Volume *23*

Supplemental Information

Insights into Collagen Uptake by C-type Mannose Receptors from the Crystal Structure of Endo180 Domains 1–4

Patricia Paracuellos, David C. Briggs, Federico Carafoli, Tan Lončar, and Erhard Hohenester

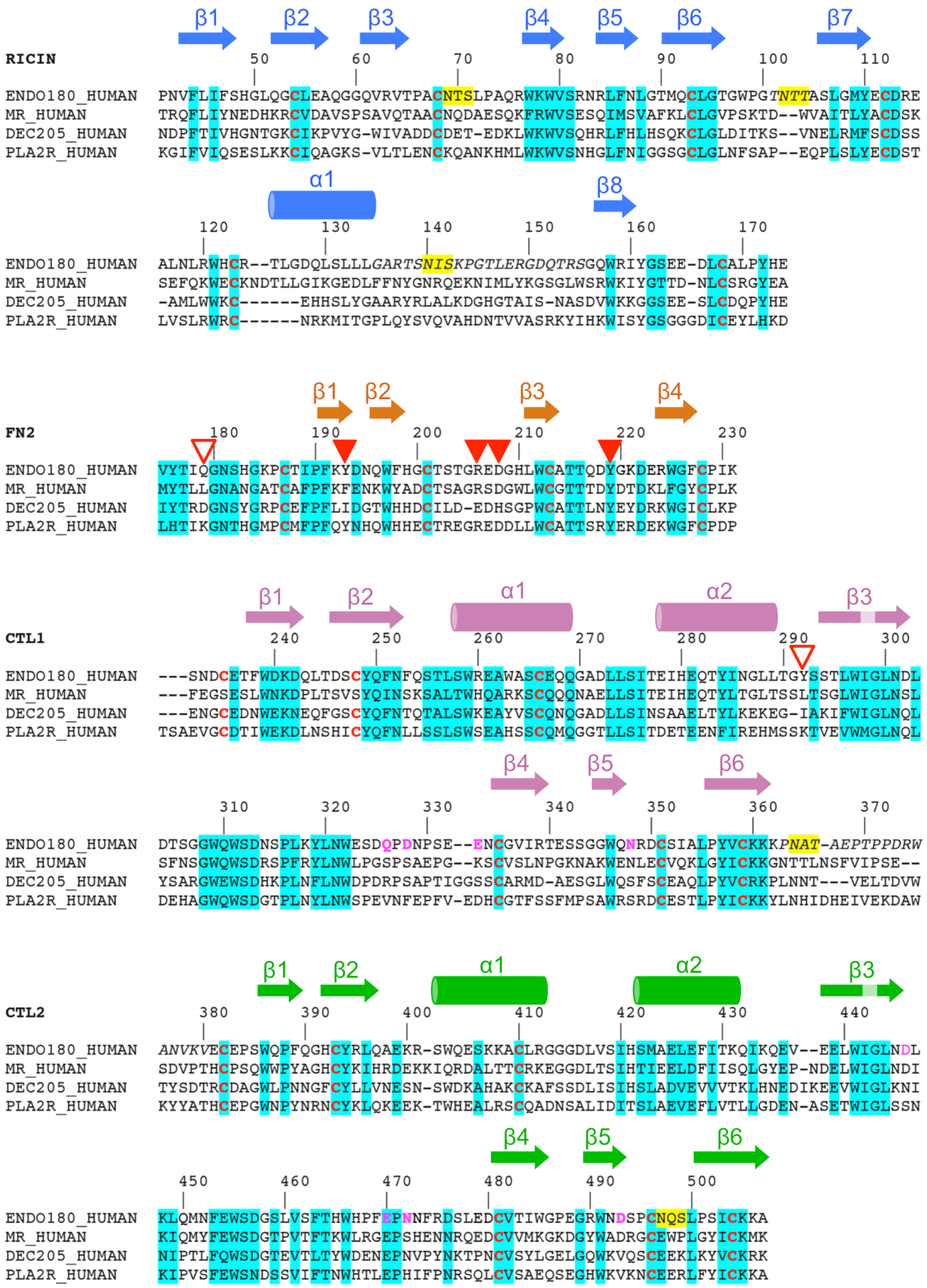

Figure S1, related to Figure 1. Sequence alignment of the D1-4 regions of human MR, Endo180, PLA$_2$R and DEC-205. The Endo180 sequence numbering and secondary structure elements are indicated above the alignment. The sequences of disordered regions in the Endo180 crystal structure are in italics. Consensus sites for *N*-linked glycosylation are highlighted in yellow. Residues involved in metal ion binding are in magenta. Residues whose mutation to alanine abolishes collagen binding are marked by filled red triangles. Positions at which an engineered glycan reduces collagen binding are marked by open red triangles.

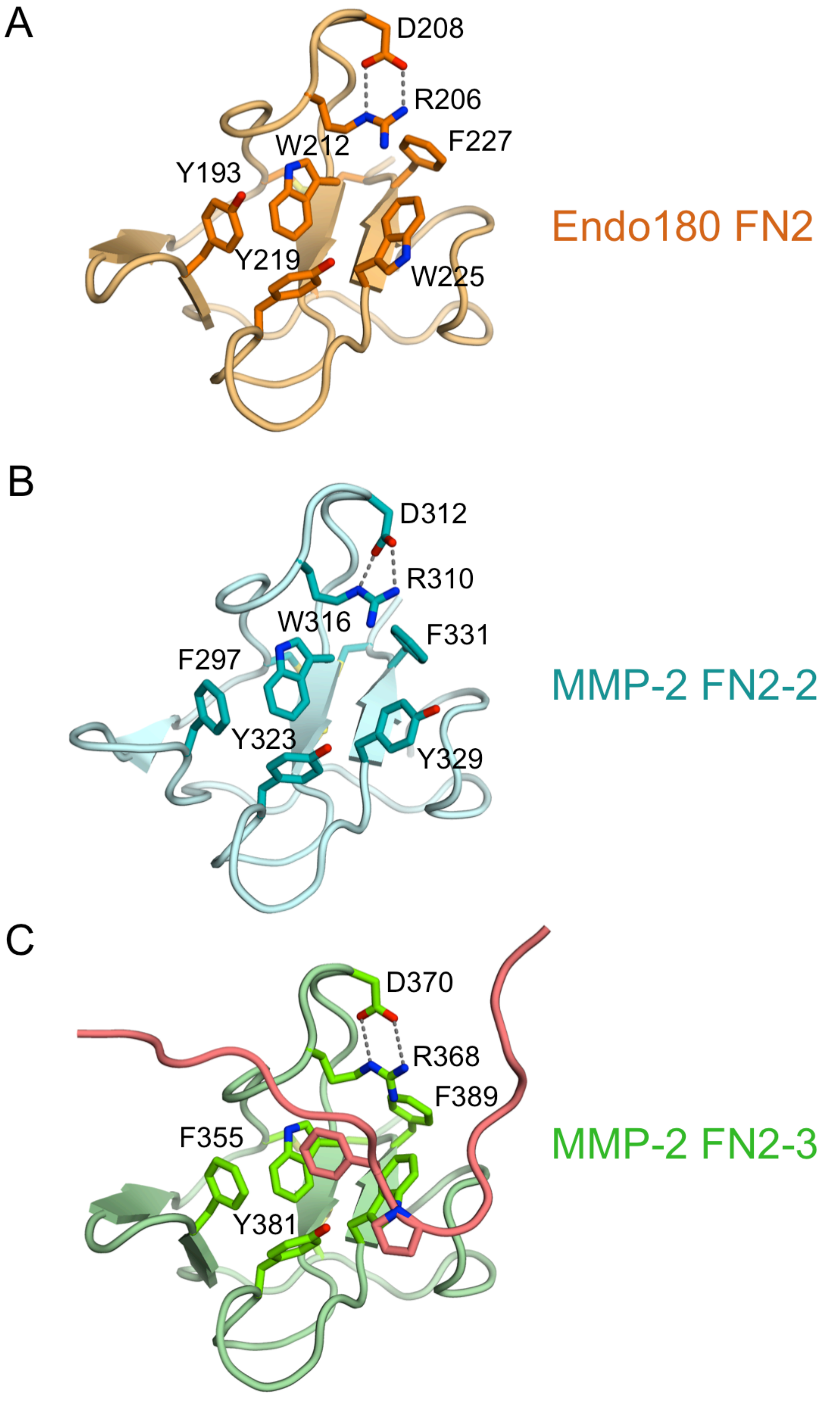

Figure S2, related to Figure 1. Comparison of the FN2 domain of Endo180 and the second and third FN2 domains of MMP-2 (Morgunova et al., 1999). Selected conserved residues are shown as sticks and labelled. In the pro-MMP-2 crystal structure, the propeptide (shown in salmon) interacts with the third FN2 domain in a manner believed to mimic the binding of gelatin.

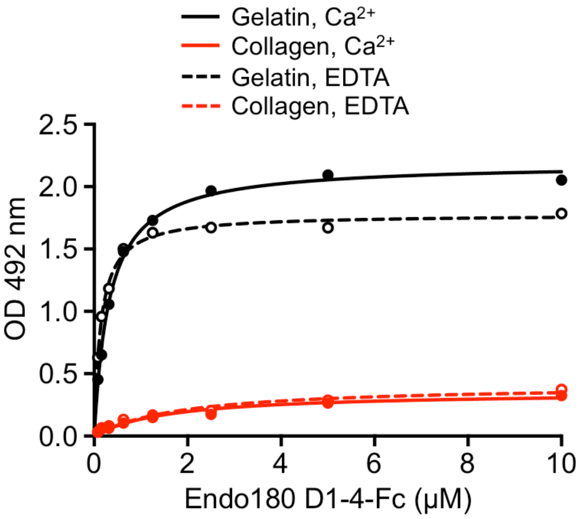

Figure S3, related to Figure 5. Solid-phase assay of Endo180 D1-4-Fc binding to immobilised type I collagen and gelatin (heat-denatured type I collagen) in the presence of 10 mM Ca^{2+} or 10 mM EDTA. The data shown are representative of two independent experiments carried out in duplicate.

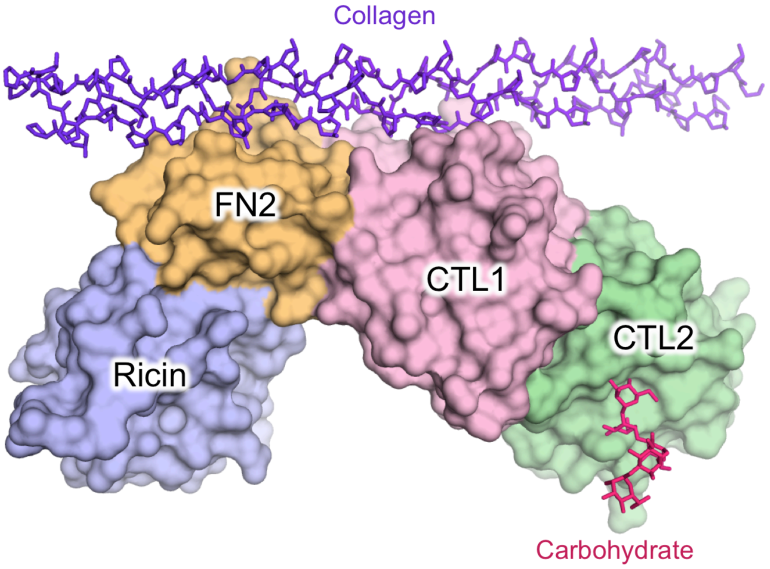

Figure S4, related to Figure 6. Model of a collagen triple helix bound to Endo180 D1-4. An idealised poly-(proline-hydroxyproline-glycine) triple helix was docked using ZDOCK (Pierce et al., 2014). The potential position of carbohydrate bound to CTL2 was obtained from the comparison with mannose-binding protein (Figure 3A).

Reference

Pierce, B.G., Wiehe, K., Hwang, H., Kim, B.H., Vreven, T., and Weng, Z. (2014). ZDOCK server: interactive docking prediction of protein-protein complexes and symmetric multimers. Bioinformatics *30*, 1771-1773.
